# A Decision-Theoretic Model of Behavior Change

**DOI:** 10.3389/fpsyg.2019.01042

**Published:** 2019-05-21

**Authors:** Kaosu Matsumori, Kazuki Iijima, Yasuharu Koike, Kenji Matsumoto

**Affiliations:** ^1^Brain Science Institute, Tamagawa University Brain Science Institute, Machida, Japan; ^2^Department of Information Processing, Tokyo Institute of Technology, Yokohama, Japan; ^3^Institute of Innovative Research, Tokyo Institute of Technology, Yokohama, Japan

**Keywords:** Theory of Planned Behavior, self-efficacy, Social Cognitive Theory, expected utility theory, Markov decision process

## Abstract

Undesirable habitual or addictive behaviors are often difficult to change. The issue of “behavior change” has long been studied in various research fields. Several models for behavior change have converged to the hypothesis that attitudes, norms, and self-efficacy are important determinants of intentions and behavior. To improve the accuracy of behavior-change models, some researchers have tried to combine behavioral economics models with existing models for behavior change. However, these attempts have failed because the existing models [e.g., Theory of Planned Behavior (TPB)] are not consistent with Expected Utility Theory (EUT), which underlies various behavioral economics models. In the present paper, we clarify the corresponding components between existing models for behavior change and EUT, and propose a new model, the Decision-Theoretic Model of behavior change (DTM), which is a natural extension of ordinary EUT.

## Introduction

It is often difficult for clinicians, trainers, or teachers to change people's undesirable habitual or addictive behaviors, such as overeating, excessive drinking, lack of exercise, and smoking. How can we help them change people's behavior for the better? The problem of “behavior change” has long been studied in various research fields such as psychology, pedagogy, nursing, public health, medicine, and health promotion (Fishbein and Ajzen, 2010). Several models for behavior change have converged to the hypothesis that attitudes, norms, and self-efficacy are important determinants of intentions and behavior (Sheeran et al., 2016). However, existing models for behavior change, such as “Social Cognitive Theory” and “Theory of Planned Behavior (TPB)” cannot sufficiently predict the occurrence probabilities of a considered behavior or its change through interventions (Sniehotta et al., 2014).

To improve the accuracy of predictive models for behavior change, some researchers have started to try to combine behavioral economics models with existing models for behavior change (Roberto and Kawachi, 2015). Because behavioral economics models consider various behavioral biases that affect the occurrence of a target behavior and/or its change through interventions, this combination was expected to be useful. However, existing models of behavior change are not consistent with Expected Utility Theory (EUT), which underlies a variety of behavioral economics models (Kahneman and Tversky, 1979; Schoemaker, 1982), and, therefore, this combination of models has been challenging.

**Figure 1:**
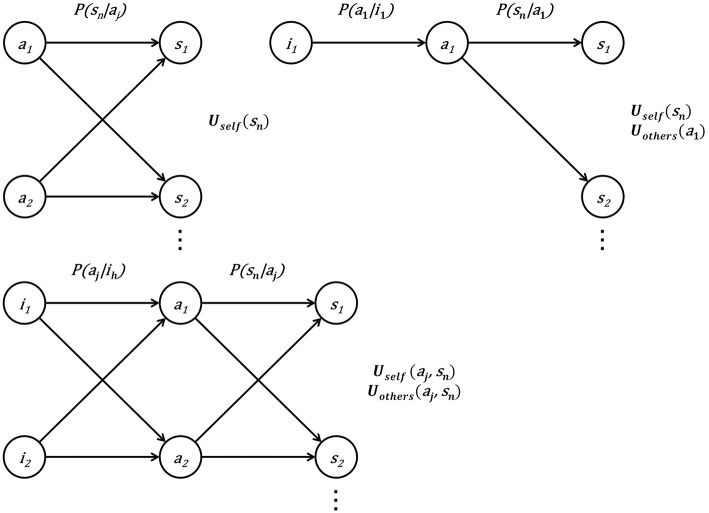
**(A)** EUT. EUT is one of the most popular approaches for rational decision-making in a stochastic environment. An action set (A = {a_1_: performing the target behavior, a_2_: not performing the target behavior}) and a state set (S = {s_1_, s_2_, …}) are assumed. The agent holds the belief that each action causes any state with a certain probability in the corresponding action-state link (P(s_n_|a_j_)). When an action a_j_ is given, the expected value of subjective utility (E[U_self_|a_j_]) is calculated. EUT states that the agent chooses a_j_, so as to maximize E[U_self_|a_j_]. **(B)** EUT-like schema of TPB. Intention to perform the target behavior (i_1_) is additionally assumed. In TPB, the three determinants of the behavioral intention are attitude toward the behavior, subjective norm, and perceived self-efficacy. The attitude toward the behavior depends on P(s_n_|a_1_) and U_self_(s_n_), subjective norms appear as U_others_(a_1_), and perceived self-efficacy appears as P(a_1_|i_1_). **(C)** DTM. The intention set I = {i_1_: intention to perform the target behavior, i_2_: intention not to perform the target behavior} as well as the action set, and the state set are assumed. The agent holds the belief that each intention causes both actions with certain probabilities of the corresponding intention-action links (P(a_j_|i_h_)) in the same way as each action causes the states with certain probabilities of the corresponding action-state links (P(s_n_|a_j_)). When i_h_ is given, the expected value of subjective utility (E[(U_self_ + wU_others_)|i_h_]) is calculated, where w denotes the weight of U_others_ relative to U_self_ in calculating subjective utility. DTM states that the agent chooses intention i_h_ so as to maximize E[(U_self_ + wU_others_)|i_h_].

In the present paper, by clarifying the corresponding components between TPB and EUT, we propose a new model, Decision-Theoretic Model of behavior change (DTM), which is consistent with EUT (Figure 1). Specifically, in DTM, we add the components of subjective norm and self-efficacy to the ordinary EUT.

In the following sections, we first explain the details of EUT; second, we explain the details of TPB and reinterpret TPB in a decision-theoretic way; third, we describe our new model as a natural extension of EUT; fourth, we discuss the superiority of DTM; and finally, we summarize our arguments and discuss future research directions.

## Expected Utility Theory (EUT)

EUT is one of the most popular approaches for rational decision-making in a stochastic environment (von Neumann and Morgenstern, 1947). When the state set (S = {s_1_, s_2_, …, s_n_, …, s_N_}), the action set (A = {a_1_, a_2_, …, a_j_, …, a_J_}), the subjective probability of a state s_n_ given an action a_j_ (P(s_n_|a_j_)), and the subjective utility of a state s_n_ (U_self_(s_n_)) are given, EUT states that the agent chooses an action a_j_ so as to maximize the expected value of subjective utility.

(1) $$E[U_{self}|a_{j}]=\sum_{n=1}^{N}P(s_{n}|a_{j})U_{self}(s_{n})$$

In the present paper, we consider a case wherein the action set has two complementary elements (A = {a_1_: performing the target behavior, a_2_: not performing the target behavior}) (Figure 1A). In many empirical studies, it is assumed that the agent's action-selection rule is based on a sigmoidal function, e.g., the logistic function (Luce, 1959; Sutton and Barto, 1998).

(2) $$P\left(a_{1}\right)=sigmoid(\beta_{1}\cdot \{E\left[U_{self}|a_{1}\right]-E\left[U_{self}|a_{2}\right]\}+\beta_{0})$$

where the inverse temperature β_1_ denotes randomness of action selection, and the constant term β_0_ denotes decision bias.

For example, consider the case with S = {s_1_: health, s_2_: disease}, A = {a_1_: exercising, a_2_: not exercising}, and that the agent has the beliefs of P(s_1_| a_1_) = 0.8, P(s_1_| a_2_) = 0.2, U_self_(s_1_) = 1, and U_self_(s_2_) = 0. Then, the expected utilities of each action are:

$$\begin{array}{l} E[U_{self}|a_{1}]=\sum_{n=1}^{2}P(s_{n}|a_{1})U_{self}\left(s_{n}\right)=0.8\cdot 1+0.2\cdot 0=0.8\\ \;E[U_{self}|a_{2}]=0.2\cdot 1+0.8\cdot 0=0.2 \end{array}$$

When the agent's internal decision parameter β_1_ = 1, and constant term β_0_ = 0, EUT predicts that P(a_1_) ≒ 0.65 in this simple situation.

## Theory of Planned Behavior

**Figure 2:**
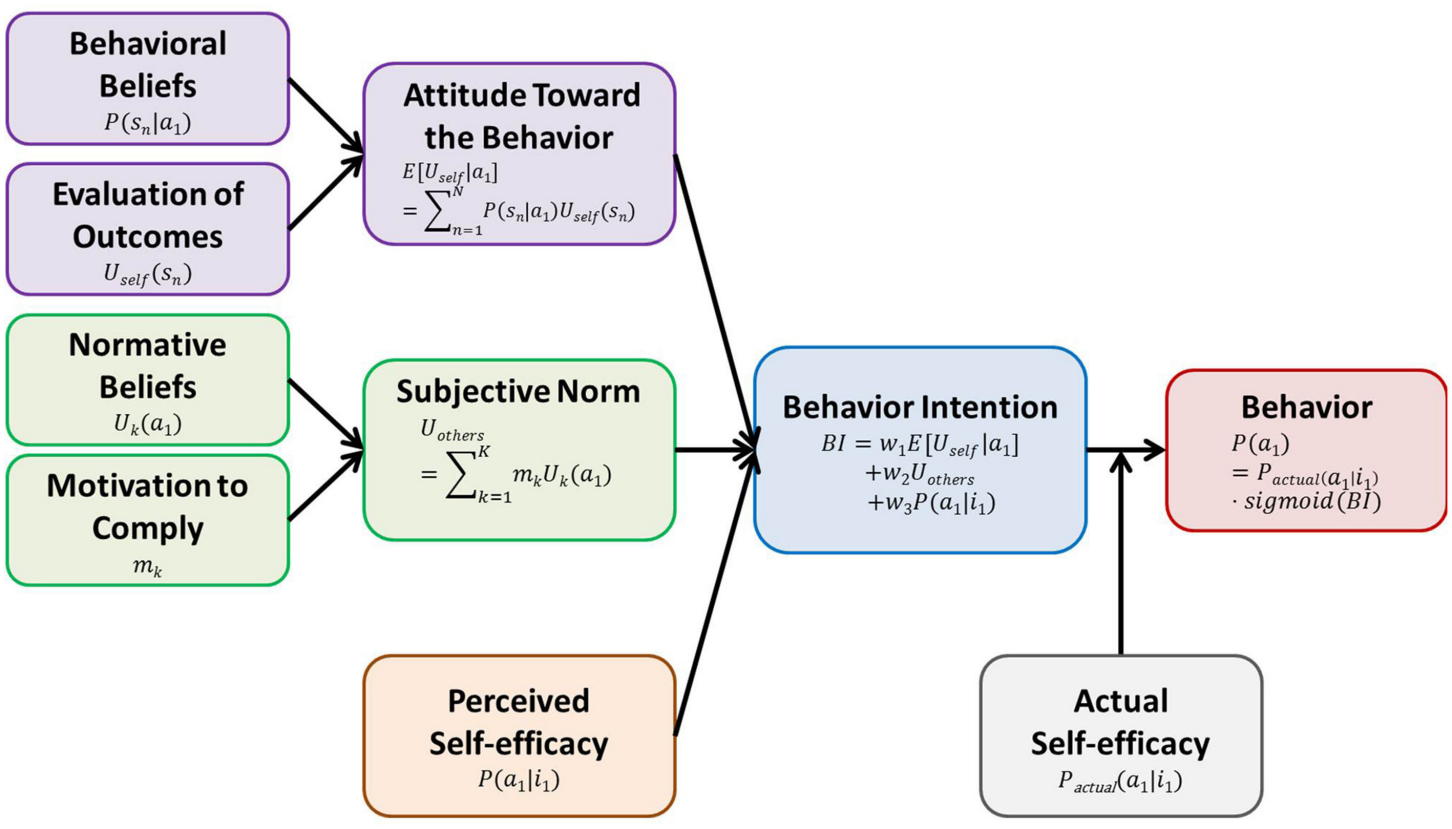
TPB. TPB is a typical model for behavior change, in which the BI for the target behavior is determined by three factors: attitude toward the behavior, subjective norm, and perceived self-efficacy. Attitude toward the behavior (E[U_self_|a_1_]) is determined by aggregating the products of each behavioral belief strength (P(s_n_|a_1_)), and evaluation of each outcome (U_self_(s_n_)) (violet). Subjective norm (U_others_) is determined by aggregating the products of each normative belief (U_k_(a_1_)), and motivation to comply (m_k_)) (green). Perceived self-efficacy is the belief about the probability of performing the target behavior successfully when the agent intends to perform it (P(a_1_|i_1_)) (orange). BI (blue) is determined by the weighted sum of attitude toward the behavior, subjective norm, and perceived self-efficacy. Occurrence of the behavior (red) is a function of BI and actual self-efficacy (P_actual_(a_1_|i_1_)) (gray).

TPB is a typical model for behavior change, in which the behavioral intention (BI) for the target behavior (a_1_) is determined by three factors: attitude toward the behavior, subjective norm, and perceived self-efficacy (Figure 2). At first glance, perceived self-efficacy is different from “perceived behavioral control,” which is the third factor of the original version of TPB, but these two concepts are treated as being the same in a newer version (Fishbein and Cappella, 2006). All behavior determinants are measured by questionnaire ratings for the target behavior. Table 1 shows the typical TPB questionnaire in the case that the target behavior is “Exercising for at least 20 min, three times per week for the next 3 months” (Fishbein and Ajzen, 2010).

**Table 1:** A typical questionnaire for TPB.

Behavioral beliefs	P(s_1_|a_1_)	My exercising for at least 20 min, three times per week for the next 3 months will result in my having a fast recovery from my surgery. likely:___1__:___2__:___3__:___4__:___5__: unlikely
Evaluation of outcomes	U_self_(s_1_)	My having a fast recovery from my surgery is good:___1__:___2__:___3__:___4__:___5__: bad
Normative beliefs	U_others_(a_1_)	My doctor thinks that my exercising for at least 20 min, three times per week for the next 3 months is good:___1__:___2__:___3__:___4__:___5__: bad
Motivation to comply	m_1_	When it comes to matters of health, I want to do what my doctor thinks I should do. Agree:___1__:___2__:___3__:___4__:___5__: disagree
Perceived self-efficacy	P(a_1_|i_1_)	I am confident that if I wanted to I could exercise for at least 20 min, three times per week for the next 3 months. Definitely true:__1__:__2__:__3__:__4__:__5__: definitely false
Behavioral intention	BI(i_1_)	I intend to exercise for at least 20 min, three times per week for the next 3 months. Likely:___1__:___2__:___3__:___4__:___5__: unlikely
Behavior	P(a_1_)	In the past 3 months, I have exercised for at least 20 min, three times per week. (This question needs to be answered 3 months after the previous questions.) true:___1__:___2__: false

*The target behavior “Exercising for at least 20min, three times per week for the next 3 months” is considered in this example. Table 1 adapted from Fishbein and Ajzen, 2010 with permission of Taylor and Francis Group LLC Books*.

Attitude toward the behavior is the agent's positive or negative evaluation of performing the target behavior a_1_ (Ajzen, 1991; Fishbein and Ajzen, 2010), which is based on EUT in economics, or expectancy-value theory in psychology (Edwards, 1954; Ajzen, 1985). Attitude toward the behavior is determined by aggregating the products of behavioral beliefs and the evaluation of outcomes. As a behavioral belief it is the belief (*subjective* probability) that performing the target behavior (a_1_) will lead to a particular outcome state (s_n_) among the state set, we consider and denote the behavioral belief as P(s_n_|a_1_) (Ajzen, 1985). As the evaluation of an outcome is the expectation of an agent's utility when the outcome is obtained, we denote it as U_self_(s_n_) (Ajzen, 1985). Then, importantly, we can consider the attitude toward the behavior as the expected utility when a_1_ is given (E[U_self_|a_1_] = Σ_n = 1_^N^P(s_*n*_|a_1_)^*^U_self_(s_*n*_)) (Edwards, 1954; Ajzen, 1985; Fishbein and Ajzen, 2010). It is worth noting that both E[U_self_|a_1_] and E[U_self_|a_2_] are considered in EUT, but only E[U_self_|a_1_] is considered in TPB.

Because the agent's behavior could not be explained well merely by attitude toward the behavior, TPB has added two other factors, subjective norm and perceived self-efficacy.

**Figure 3:**
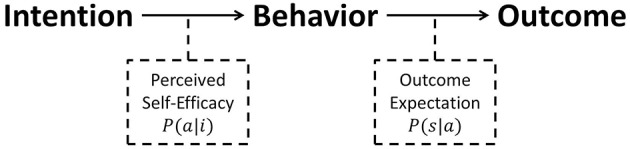
Bandura's schema. Perceived self-efficacy as well as outcome expectation are considered as determinants of human behavior. Perceived self-efficacy (P(a|i)) is the belief about the probability of performing the behavior successfully when the agent intends to perform it. Outcome expectation (P(s|a)) is the belief about the probability of a particular outcome, given the agent's target behavior.

Subjective norm is the perceived social pressure to engage or not engage in a behavior (Fishbein and Ajzen, 2010). Subjective norm is determined by aggregating the products of normative beliefs and the motivation to comply with other individuals (*m*_k_; k = 1, 2, …, K). As normative beliefs refer to the agent's belief about the degree to which a particular individual, K, thinks the agent should perform the target behavior a_1_, we consider it as the agent's expectation of the individual's utility when the target behavior is performed, and denote it as U_k_(a_1_). Then, we can consider the subjective norm as the weighted sum of other individuals' utilities (U_others_(a_1_) = Σ_k = 1_^K^${\text{m}}_{\text{k}}^{*}$U_k_(a_1_)) (Fishbein and Ajzen, 2010). It is worth noting that other individuals' utilities are a function of action, whereas the agent's utility in attitude toward the behavior is a function of state, in TPB.

(Perceived) self-efficacy, originally proposed by Bandura (Bandura, 1977), is a personal judgement of “how well one can execute courses of action required to deal with prospective situations” (Bandura, 1982). Bandura emphasized it as a determinant of human behavior in addition to outcome expectations (Figure 3). As perceived self-efficacy for the target behavior a_1_ is the belief about the probability of performing the behavior successfully when the agent intends to perform the target behavior (i_1_), we denote it as P(a_1_|i_1_). It is worth noting here that the outcome expectation corresponds to the behavioral beliefs mentioned above, because it is defined as an agent's estimate that a given behavior will lead to certain outcomes.

The weighted sum of these three determinants—attitude toward the behavior, subjective norm, and perceived self-efficacy—determines BI (Figure 2).

(3) $$BI\left(i_{1}\right)=w_{1}E[U_{self}|a_{1}]+w_{2}U_{others}\left(a_{1}\right)+w_{3}P(a_{1}|i_{1})$$

where, w_1_, w_2_, and w_3_ denote the weight of attitudes toward the behavior, subjective norm, and perceived self-efficacy, respectively. This equation can be simplified to:

(3′) $$\begin{array}{l} BI\left(i_{1}\right)=w_{3}P\left(a_{1}|\;i_{1}\right)\\ \;+\sum_{n=1}^{N}P\left(s_{n}|\;a_{1}\right)\{w_{1}U_{self}\left(s_{n}\right)+w_{2}U_{others}\left(a_{1}\right)\} \end{array}$$

which allows us to compare it with DTM later [section Decision-Theoretic Model of Behavior Change (DTM)]. The second term of Equation 3 and the corresponding part of Equation 3′ about U_others_ are equivalent, because $\overset{N}{\underset{n=1}{\sum }}P\left(s_{n}|a_{1}\right)=\;1.$

Here, we note that BI is not consistent with EUT, because subjective norm and perceived self-efficacy are simply added to E[U_self_|a_1_]. In other words, attempts to improve the model's accuracy by incorporating subjective norm and perceived self-efficacy in TPB are inconsistent with EUT, which underlies a variety of behavioral economics models (Kahneman and Tversky, 1979; Schoemaker, 1982). We tried to draw a schematic view of TPB while maintaining consistency with EUT, as much as possible (Figure 1B). In the EUT-like schema of TPB, the three determinants of behavioral intention can be identified. However, their summation does not mathematically provide the occurrence probability of the target behavior in the EUT-like schema.

When the target behavior is considered as a dichotomous variable ({a_1_: performing the target behavior, a_2_: not performing the target behavior}), logistic regression is commonly used to predict the agent's intention. This corresponds to the assumption that the agent's intention-selection rule is based on a sigmoidal function, e.g., the logistic function Luce, 1959; Sutton and Barto, 1998.

(4) $$P\left(i_{1}\right)=sigmoid(\beta_{1}\cdot BI\left(i_{1}\right)+\beta_{0})$$

The occurrence probability of the target behavior (P(a_1_)) is a function of P(i_1_) and actual (not perceived) self-efficacy. As actual self-efficacy for the target behavior a_1_ should be the objective probability of performing the behavior successfully when the agent intends to perform a_1_, we denote it as P_actual_(a_1_|i_1_). However, in many cases, actual self-efficacy is difficult to measure through questionnaires. In such cases, perceived self-efficacy is used as a proxy for actual self-efficacy. Then, the estimated occurrence probability of the target behavior is:

(5) $$P\left(a_{1}\right)=P_{actual}\left(a_{1}|i_{1}\right)\cdot P\left(i_{1}\right)≒P\left(a_{1}|i_{1}\right)\cdot P\left(i_{1}\right)$$

Here, note that the TPB questionnaire (Table 1) does not include any questions regarding the belief about the probability of achieving the target behavior (a_1_) when the agent intends not to perform the behavior (i_2_). Calculating P(a_1_) without considering P(a_1_|i_2_) (≒P_actual_(a_1_|i_2_)) is allowed when P(a_1_|i_2_) is assumed to be zero, which enables us to calculate P(a_1_) just with P_actual_(a_1_|i_1_) and P(i_1_) (cf. Equation 8).

Thus, P(a_1_), which requires the value of P(i_1_) based on BI(i_1_) to be calculated, is what researchers would like to predict in behavior change studies. Therefore, typical TPB questionnaires contain questions about P(s_n_|a_1_), U_self_(s_n_), U_k_(a_1_), m_k_, and P(a_1_|i_1_), to predict P(a_1_) (Table 1).

## Decision-Theoretic Model of Behavior Change (DTM)

As we mentioned in the Introduction, some researchers recently tried to combine behavioral economics models with existing models for behavior change (Roberto and Kawachi, 2015) to improve the accuracy of the prediction of behavior. However, the existing models of behavior change challenge this combination, because they are not consistent with EUT.

Here, we propose a new model, DTM, which is consistent with EUT. In DTM, we add the components of subjective norm and self-efficacy to the ordinary EUT. To do so, we introduce an intention set (I = {i_1_: intention to perform the target behavior, i_2_: intention not to perform the target behavior}), in addition to the state set (S = {s_1_, s_2_, …, s_n_, …,s_N_}) and the action set (A = {a_1_: performing the target behavior, a_2_: not performing the target behavior}), which were already included in EUT (Figure 1C).

The occurrence of i_h_ (h = 1, 2) is determined by expected utility (E[U_total_|i_h_]) in DTM. E[U_total_|i_h_] is an aggregation of the products of the subjective probability of a state s_n_ given an intention i_h_ (P(s_n_|i_h_) = Σ_j = 1_^2^P(s_n_|a_j_)^*^P(a_j_|i_h_)), and the total utility of a state (U_total_(s_n_)). We assume that total utility is a summation of the agent's utility and others' utility (U_total_ = U_self_ + wU_others_), both of which are functions of state and behavior, where w denotes the weight of U_others_ relative to U_self_ in calculating subjective utility. Thus, expected utility E[U_total_|i_h_] is:

(6) $$\begin{array}{l} E\left[U_{total}|i_{h}\right]\\ =\sum_{j=1}^{2}P(a_{j}|i_{h})\sum_{n=1}^{N}P(s_{n}|a_{j})\{U_{self}\left(a_{j},\;s_{n}\right)+wU_{others}\left(a_{j},\;s_{n}\right)\} \end{array}$$

Note that other individuals' utilities in subjective norm are functions of action, whereas the agent's utility in attitude toward the behavior is a function of state in TPB. Here, in DTM, we defined both the agent and other individuals' utilities as functions of action and state.

To compare with TPB, we denote equation 6 as follows:

(6′) $$\begin{array}{l} E\left[U_{total}|i_{h}\right]\\ =P(a_{1}|i_{h})\sum_{n=1}^{N}P(s_{n}|a_{1})\{U_{self}\left(a_{1},\;s_{n}\right)+wU_{others}\left(a_{1},\;s_{n}\right)\}\\ +P\left(a_{2}|\;i_{h}\right)\sum_{n=1}^{N}P\left(s_{n}|\;a_{2}\right)\{U_{self}\left(a_{2},\;s_{n}\right)+wU_{others}\left(a_{2},\;s_{n}\right)\} \end{array}$$

Equation 3′ of TPB and Equation 6′ of DTM are different in the following five ways (Figures 1B,C):

(1)E[U_total_|i_1_] is a kind of expected utility; E[U_total_|i_1_] in DTM is naturally extended from E[U_self_|a_1_] in EUT by adding the components of subjective norm and perceived self-efficacy. In contrast, BI(i_1_) in TPB cannot be considered as expected utility.
(2) DTM considers not only the expected utility given i_1_ (E[U_total_|i_1_]), but also the expected utility given i_2_ (E[U_total_|i_2_]), whereas TPB considers behavioral intention only for i_1_ (BI(i_1_)). This difference is important when we consider P(i_1_) and P(a_1_) later in this section.
(3) U_self_(a_j_, s_n_) and U_others_(a_j_, s_n_) in DTM are more flexible functions than U_self_(s_n_) and U_others_(a_1_) in TPB. TPB cannot consider cases in which the agent's utility depends on his/her action cost, or other individuals' utilities depend on the consequences of their actions.
(4) E[U_total_|i_1_] in DTM considers the utility of the case in which the agent intends to perform the target behavior (i_1_), but fails to perform it and instead, performs an alternative action (a_2_). However, BI(i_1_) in TPB cannot take this into account.
(5) Perceived self-efficacy (P(a_j_|i_h_)) is *multiplied* by expected utility given an action in DTM but is *added* to expected utility given a_1_ in TPB.

We assume that the intention-selection rule is based on the sigmoidal function, as with EUT Luce, 1959; Sutton and Barto, 1998.

(7) $$P\left(i_{1}\right)=sigmoid(\beta_{1}\cdot \{E\left[U_{total}|i_{1}\right]-E\left[U_{total}|i_{2}\right]\}+\beta_{0})$$

The difference between Equation 4 (TPB) and 7 (DTM) is that E[U_total_|i_2_] is explicitly considered in Equation 7, but not in Equation 4. This difference is not important when E[U_total_|i_2_] is stable across subjects or contexts, because it is adsorbed into a constant term. If E[U_total_|i_2_] varies across subjects or contexts, which should be a plausible assumption, it significantly affects P(i_1_).

The estimated occurrence probability of the target behavior is:

(8) $$\begin{array}{l} P\left(a_{1}\right)=P_{actual}\left(a_{1}|i_{1}\right)\cdot P\left(i_{1}\right)+P_{actual}\left(a_{1}|i_{2}\right)\cdot P\left(i_{2}\right)\\ \;≒P\left(a_{1}|i_{1}\right)\cdot P\left(i_{1}\right)+P\left(a_{1}|i_{2}\right)\cdot P\left(i_{2}\right) \end{array}$$

The difference between Equation 5 (TPB) and Equation 8 (DTM) is that Equation 8 explicitly considers the case in which the agent performs the target behavior despite the absence of an intention to do so. This difference is not important only if P_actual_(a_1_|i_2_) and/or P(i_2_) are zero, because Equation 5 (TPB) and Equation 8 (DTM) are the same in this case.

Thus, the occurrence probability of the target behavior is predicted by using these equations (Equations 6–8) in DTM. Therefore, DTM needs some additional questions in its questionnaires (Table 2).

**Table 2:** A proposed questionnaire for DTM.

Behavioral beliefs	P(s_1_|a_2_)	NOT exercising for at least 20 min, three times per week for the next 3 months, will result in a fast recovery from my surgery. likely:___1__:___2__:___3__:___4__:___5__: unlikely
Perceived self-efficacy	P(a_1_|i_2_)	I am confident that even if I DO NOT want to, I could exercise for at least 20 min, three times per week, for the next 3 months. definitely true:__1__:__2__:__3__:__4__:__5__: definitely false
Evaluation of outcomes	U_self_(a_1_, s_1_)	My exercising for at least 20 min, three times per week for the next 3 months AND having a fast recovery from my surgery are good:___1__:___2__:___3__:___4__:___5__: bad
Evaluation of outcomes	U_self_(a_1_, s_2_)	My exercising for at least 20 min, three times per week for the next 3 months AND NOT having a fast recovery from my surgery are good:___1__:___2__:___3__:___4__:___5__: bad
Evaluation of outcomes	U_self_(a_2_, s_1_)	My NOT exercising for at least 20 min, three times per week for the next 3 months AND having a fast recovery from my surgery are good:___1__:___2__:___3__:___4__:___5__: bad
Evaluation of outcomes	U_self_(a_2_, s_2_)	My NOT exercising for at least 20 min, three times per week for the next 3 months AND NOT having a fast recovery from my surgery are good:___1__:___2__:___3__:___4__:___5__: bad
Normative beliefs	U_others_(a_1_, s_1_)	My doctor thinks that my exercising for at least 20 min, three times per week for the next 3 months AND having a fast recovery from my surgery are good:___1__:___2__:___3__:___4__:___5__: bad
Normative beliefs	U_others_(a_1_, s_2_)	My doctor thinks that my exercising for at least 20 min, three times per week for the next 3 months AND NOT having a fast recovery from my surgery are good:___1__:___2__:___3__:___4__:___5__: bad
Normative beliefs	U_others_(a_2_, s_1_)	My doctor thinks that my NOT exercising for at least 20 min, three times per week for the next 3 months AND my having a fast recovery from my surgery are good:___1__:___2__:___3__:___4__:___5__: bad
Normative beliefs	U_others_(a_2_, s_2_)	My doctor thinks that my NOT exercising for at least 20 min, three times per week for the next 3 months AND NOT having a fast recovery from my surgery are good:___1__:___2__:___3__:___4__:___5__: bad

*A typical questionnaire, which is modified from that for TPB, is shown with the same example (exercise after surgery) as Table 1*.

To summarize, DTM is a natural extension of EUT, which accounts for behavior change.

## An Example Showing the Superiority of DTM

Here, we focus on the fifth difference between Equations 3′ and 6′ in section Decision-Theoretic Model of Behavior Change (DTM), to assert the superiority of DTM over TPB. Whereas, perceived self-efficacy is multiplied by the weighted sum of attitude toward the behavior and subjective norm in DTM (Equation 6′), it is added to these factors in TPB (Equation 3′), as we noted above.

Let us think about the case of opening a tight jar lid. For the sake of simplicity, let us assume that there is no other individual present. The target behavior (a_1_) is “straining the wrist enough to open the jar lid.” Here, i_1_ is “intention to strain the wrist enough to open the jar lid,” s_1_ is “the lid was opened,” and s_2_ is “the lid was not opened.”

In TPB, BI is determined by the following factors: (1) Attitude toward the behavior, which is governed by the value of the contents of the jar to oneself, (2) Subjective norm, which can be ignored in this case, because the absence of any other individual is assumed, (3) Perceived self-efficacy, which is the belief about the probability of straining the wrist enough to open the jar lid when one intends to do it. The estimated weight for attitude toward the behavior, and that for perceived self-efficacy are assumed to be positive in this case. Now, let us assume that this person injured his/her spinal cord and became totally paralyzed. Then, perceived self-efficacy would change to 0, but the attitude toward the behavior (or the subjective norm) would not change. Because BI of TPB is determined by the weighted sum of the attitude toward the behavior, the perceived self-efficacy, and the subjective norm (ignored here), TPB would predict that one will have the intention to strain the wrist enough to open the jar lid, regardless of her/his inability to move, in proportion to the value of the contents of the jar. This prediction is unrealistic, thus presenting a counterexample for TPB.

In contrast, DTM can properly predict that BI is consistently zero regardless of the value of the contents, because the weighted sum of attitude toward the behavior (and the subjective norm) is multiplied by perceived self-efficacy (= 0), showing the superiority of DTM.

## Discussion

In the present paper, we show that TPB could be considered as an attempt to improve the EUT's accuracy of predicting behavior change, by incorporating subjective norm and self-efficacy. Indeed, TPB has achieved great success, because it is a relatively simple model, and its three factors are actually effective in promoting behavior change (Sheeran et al., 2016). Applying TPB has allowed investigators to identify important psychological factors to understand, predict, and change human social behavior (Van Lange et al., 2011). Moreover, behavior change interventions applying TPB were actually effective in two-thirds of studies (Hardeman et al., 2002), indicating that TPB is appropriate for clinical application.

However, TPB has a serious problem. Because subjective norm and perceived self-efficacy are simply added to the standard expected utility in TPB, it is not consistent with EUT, and thus, cannot be connected with behavioral economics models. To overcome this problem, we propose a new behavior change model, DTM, which includes the components of subjective norm and self-efficacy as a natural extension of EUT.

As DTM is consistent with EUT, it can be easily extended in several ways. First, DTM can handle intertemporal choices by using temporal discounted utility. In particular, hyperbolic discounting, which is well-studied in behavioral economics, is important for behavior change because it can express procrastination (Story et al., 2014). Second, DTM can be easily extended to a Markov model by introducing a Markov decision process (MDP) framework. Markov models are useful when the situation is continuous over time, and important events may happen more than once (Sonnenberg and Beck, 1993; Sutton and Barto, 1998). Because most current neural models of the reward system are based on MDP, this extension enables us to combine behavior change models with pharmacological models of aberrant behavior such as addiction (Redish, 2004; Rangel et al., 2008). Third, we simply defined U_total_ by the weighted sum of U_self_ and U_others_ in the present paper, but other ways of formulating U_total_ are possible when considering various types of social preferences, such as inequality aversion, guilt aversion, and Rawlsian preferences (Fehr and Krajbich, 2014). Fourth, DTM could be applicable to studies about morality (Crockett, 2013). In DTM, we introduced a distinction between action and intention into the EUT, and this is an important character of moral judgement (Cushman, 2008). Utility in DTM is suitable to represent moral values, because it could be a function of not only action and outcome, but also intention [i.e., U_self_(i_h_, a_j_, s_n_), U_others_(i_h_, a_j_, s_n_)].

We hope that DTM leads to a better combination of existing models of behavior change and behavioral economics models.

## Author Contributions

KaM conceptualized the data. KaM and KeM wrote the paper. KaM, KeM, KI, and YK revised the paper.

### Conflict of Interest Statement

The authors declare that the research was conducted in the absence of any commercial or financial relationships that could be construed as a potential conflict of interest.
